# A Low-Profile SIW-Based CTS Array with Reconfigurable Four Beams and Dual Polarizations for K-Band Sensing

**DOI:** 10.3390/s22093563

**Published:** 2022-05-07

**Authors:** Yitong Jin, Yuanqing Chen, Yafei Ding, Ziwen Zou, Feng Qian, Yong Luo, Guangli Yang

**Affiliations:** 1 School of Communication & Information Engineering, Shanghai University, Shanghai 200444, China; yitong_jin@shu.edu.cn (Y.J.); 18717833950@163.com (Y.C.); yafeiding@shu.edu.cn (Y.D.); zwenzou_97@163.com (Z.Z.); hayiji2015@shu.edu.cn (F.Q.); y_luo@foxmail.com (Y.L.); 2 Suzhou Dufeng Technology Co., Ltd., Suzhou 215028, China

**Keywords:** continuous transverse stub (CTS), reconfigurable beams, millimeter-wave antennas, dual polarizations, substrate-integrated-waveguide (SIW)

## Abstract

A dual-polarized continuous transverse stub (CTS) K-band antenna with reconfigurable four beams and low profile is proposed based on substrate-integrated-waveguide (SIW) design. It consists of a line source generator (LSG) on the bottom surface, a spherical-wave to plane-wave transforming part on the middle layer, and CTS radiators on the top surface. Particularly, the LSG has four SIW-based H-plane horns, and a chip is integrated to switch among the two pairs of horns, so as to transfer the quasi-TEM waves on the bottom surface by a ±10° deflection angle to the middle layer for the CTS radiators on the top surface, resulting in four reconfigurable scanning beams with 10° for two polarizations. The measurements show that it realizes four reconfigurable beams with a 25.8 dBi gain at 24 GHz, verifying the design. The proposed antenna takes into account the advantages of reconfigurable multi-beam, dual polarization, low side lobes, low profile, and high gain, which can be applied to K-band sensing, especially for wind profile radars.

## 1. Introduction

Recently, wind profile radar is widely used in the field of climate monitoring [1], monitoring the structure of the atmospheric boundary [2], and extracting information such as wind speed and wind direction [3,4,5]. A wind profile radar antenna can usually be realized by the two methods of doppler beam steering (DBS) and spaced antenna (SA). The former determines the horizontal wind speed by controlling the antenna beam, while the latter obtains the wind speed according to the time delay between receivers [6]. In contrast, DBS is well accepted in wind profile radars due to its generally higher signal-to-noise (SNR) ratio than SA [7]. In the DBS method, large-scale phased array radar is generally used, among which, mechanical beam scanning is more common, while electronic beam scanning is rarely involved, which is expected to become a breakthrough point. In [8,9], a low-profile Ku-band phased array transmitter consisting of 256 dual-polarized antenna elements and a broadband phased array structure based on a (2 × 2) transmit/receive quad-beamforming chip have been proposed. However, a phased array antenna system needs plenty of integrated transceiver chips, and expensive and complex phase shifters, meanwhile, the isolation is not high enough and the power consumption is large as well [10].

In [11], a beam-reconfigurable antenna for unmanned aerial vehicles (UAVs) with wide beam coverage is proposed, which has a simple structure, and a limited peak gain of 5.8 dBi. Leaky-wave-based continuous transverse stub (CTS) planar array antennas can obtain beam scanning properties as well [12], and much research has been explored. A Ka-band waveguide continuous transverse stub antenna array with over 75% aperture efficiency with a low cross-polarization less than −46 dB is studied in [13], and in [14], the conventional multilayer parallel-plate waveguide (PPW) feed by a novel LSG is proposed with realizing frequency-dependent beam scanning properties.

In addition, to miniaturize the size and decrease the loss, the technology of SIW has been widely used in the design of CTS. In [15] a K-band CTS array based on a substrate-integrated waveguide with a peak gain of 22.6 dBi and a wide impendence bandwidth of 32.6% from 17.2 to 23.9 GHz was proposed. In [14], SIW technology was used, achieving a peak gain of 20.6 dBi and an antenna efficiency of over 82%. For frequency scanning, the nonuniform slow-wave structure was also used for a CTS antenna to control the amplitude and phase distributions [16]. In addition, in [17], the beam scanning of the array was realized by simply moving the small SIW horn along the line across the focal point. A novel SIW multi-beam antenna, based on the parabolic reflector principle, was proposed in [18]. This research demonstrates that an SIW-based CTS antenna can realize good frequency-dependent beam scanning properties. According to the wind profile radar scenario, not only is the antenna array required to have beam scanning characteristics, but also four-beam, dual-polarization, high gain, low side lobes, and low profile requirements are proposed, based on which, the antenna proposed in this communication is a good choice.

In this communication, a reconfigurable multi-beam SIW-CTS array is proposed by using the SIW technology and the LSG, with the characteristics of dual-polarization, high-gain and a low-sidelobe. Four beams are reconfigurable by moving the H-plane horn along the line across the focal point of the reflector in both horizontal and vertical polarizations.

## 2. SIW-Based CTS Antenna Design and Analysis

**Figure 1:**
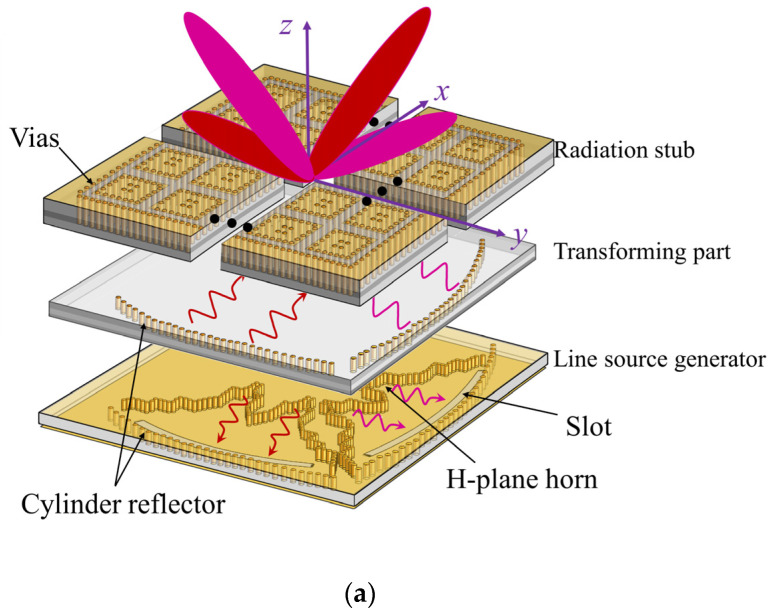

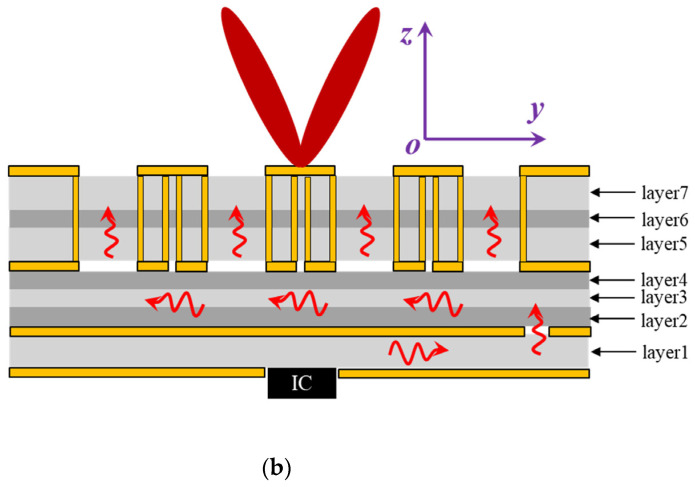
(**a**) The configuration of the proposed SIW-based CTS antenna includes three parts: the LSG part in the bottom layer, the transforming part that transfers the spherical waves to plane waves in the middle, and the leaky radiation part in the top layer. (**b**) The side view.

The proposed SIW-based CTS array antenna, as shown in Figure 1a, consists of three parts: the LSG structure in the bottom layer (layer 1), the transformation layers (layers 2–4), and the CTS radiation parts (layers 5–7). As in Figure 1b, the LSG contains four H-plane horns for generating ±45° polarizations, two parabolic cylinder reflectors and two coupling slots in the terminal for coupling generated spherical waves to the upper layers, thereby, propagating backwards and transforming the spherical waves to plane waves. Afterwards, the plane waves propagate along the 22 × 22 CTS array, and radiate to the free space. Particularly for +45° polarization, these two H-horn antennas are arranged with different positions, thus, as the waves are reflected and coupled from the bottom layer to the upper layers, phase differences are induced, leading to beam scanning in 24 GHz.

### 2.1. Line Source Generator and Transformation Part

**Figure 2:**
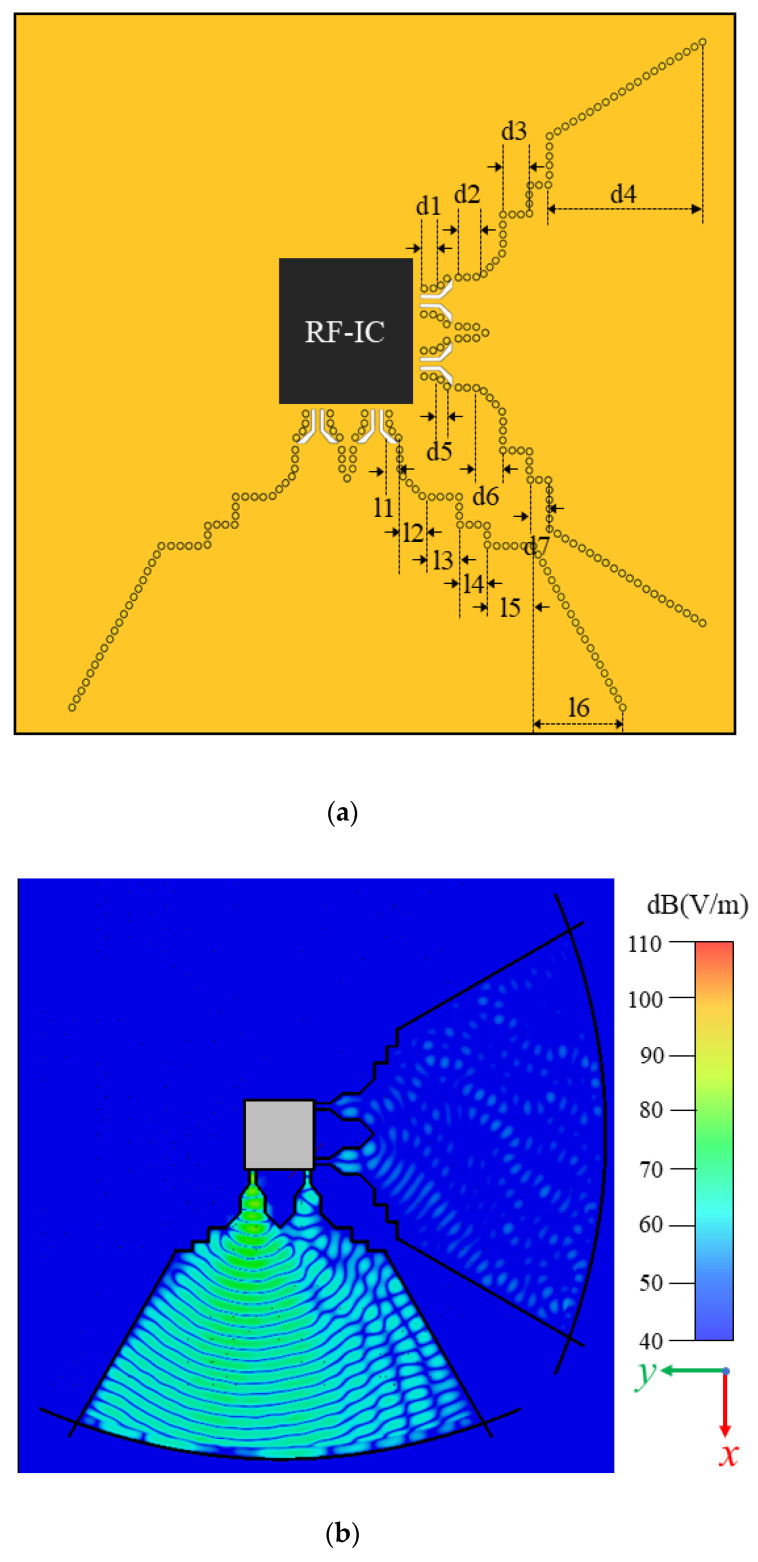
(**a**) LSG contains two H-horn antennas in y directions for +45° polarization and another two horn antennas in x direction for −45° polarization. (l1–l6 and d3–d7 are the parameters of the LSG.) (**b**) Electric-field distribution of LSG with H-plane horn at 94.7 mm away from the focal point at 24 GHz.

The line source generator (LSG) is designed to convert the TE_10_ mode from the rectangular waveguide to a quasi-TEM wave for exciting the CTS array. It consists of a substrate (Rogers 4835, $\varepsilon_{r}$ = 3.55, tanδ = 0.0037) with a grounded RF layer underneath and another metallic layer on the top, as shown in Figure 2a. The parameters of the LSG are as follows: l1 = 3 mm, l2 = 4.38 mm, l3 = 4.7 mm, l4 = 4.7 mm, l5 = 5.2 mm, l6 = 26.6 mm, d1 = 4.6 mm, d2 = 5 mm, d3 = 3.5 mm, d4 = 46 mm, d5 = 3.67 mm, d6 = 4.38 mm, d7 = 2.5 mm. The LSG contains three parts: H-plane horn, parabolic cylinder reflector, and coupling slots. In the substrate, a grounded-co-planar waveguide (GCPW) is used to excite the H-plane horn, and two gradually changed slots are designed to realize the impedance matching in the wideband, according to [19]. To avoid any bandgap and make the wave leakage loss as small as possible, the distance between two vias and the diameter can be designed according to formulas (1) and (2):

(1) $$1<p_{via}/d_{via}<2.5$$

(2) $$0.05<p_{via}/\lambda_{c}<0.25$$

where $p_{via}$ represents the spacing between vias, $d_{via}$ represents the diameter of the vias, and $\lambda_{c}$ represents the cut-off wavelength of the SIW. In addition, the arc coupling groove was designed to connect the radiator to avoid the leakage of electromagnetic energy. By simply moving the H-plane horn along the focal point in the *x*-axis, the two horn antennas have a different phase delay, and generate waves propagating in the substrate, resulting in ±10° deflection angle, as shown in Figure 2b, in which horn 1 and horn 2 were excited, respectively. By applying the generating line source feature of the horn and cylinder reflector, a long feed line source with the Taylor level distribution is proposed and designed, thereby obtaining a low sidelobe. Owing to the slight displacement between these two horn antennas that leads to the phase delay, it can obtain two scanning beams with ±10°. Similarly, another two horn antennas can obtain two scanning beams with ±10° for the orthogonal polarizations.

### 2.2. Transformation Part

**Figure 3:**
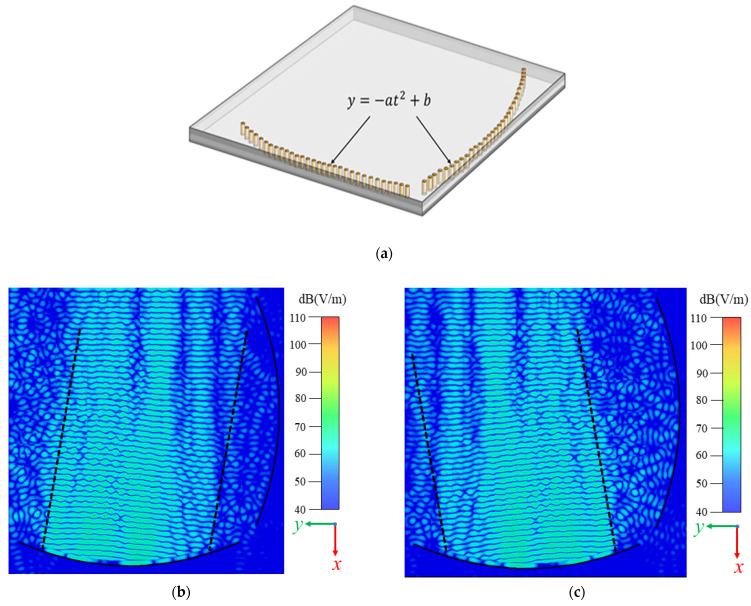
(**a**) The transforming part transfers the spherical waves generated from LSG to plane waves. Transformed spherical waves of the two H-plane horn antennas are shown in (**b**,**c**) at 24 GHz. (*a* = 0.003 mm, *b* = 94.7 mm).

As shown in Figure 3a, the transformation part has two substrates (Rogers 4450F, $\varepsilon_{r}$ = 3.7, tanδ = 0.004; Rogers 4835, $\varepsilon_{r}$ = 3.55, tanδ = 0.0037,) and consists of two reflectors in an arc shape and a line shape, respectively, for each polarization. Since the waves are generated with the TE_10_ mode from the rectangular horn antenna, it propagates as a quasi-TEM mode in the spherical form, until it is reflected and coupled to the transformation part. In the transformation part, as shown in Figure 3b, the spherical waves are transformed to plane waves, thus, it is ready to be radiated through the CTS array above. In Figure 3c, the transformed plane waves from the second excited horn antenna have a certain deflection angle, due to the displacement between this antenna with the first horn antenna that causes the phase delays.

### 2.3. Design of the Radiator

**Figure 4:**
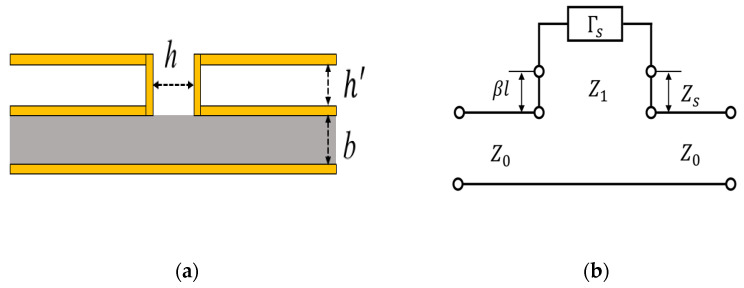
(**a**) Structure of SIW-CTS unit. (**b**) Equivalent circuit of SIW-CTS unit.

The structure and equivalent circuit of the SIW-CTS unit are shown in Figure 4, in which CTS is equivalent to a series of circuits. According to the equivalent circuit of Figure 4b, the reflection coefficient $\Gamma_{S}$ from the radiation stub can be expressed as:

(3) $$\Gamma_{s}=\frac{Z_{S}-Z_{1}}{Z_{S}+Z_{1}}$$

where $Z_{1}$ is the characteristic impedance of the radiation stub, and $Z_{S}$ is the end load impedance. Then the input impedance $Z_{se}$ can be expressed as:

(4) $$Z_{se}=Z_{1}\frac{1+\Gamma_{s}e^{-2j\beta l}}{1-\Gamma_{s}e^{-2j\beta l}}$$

(5) $$\frac{Z_{se}}{Z_{0}}=\frac{Z_{1}\left(1+\Gamma_{s}e^{-2j\beta l}\right)}{Z_{0}\left(1-\Gamma_{s}e^{-2j\beta l}\right)}$$

where βl is the electrical length of the radiation stub, and $Z_{0}$ is the characteristic impedance of the transmission line. Since $\frac{Z_{1}}{Z_{0}}=\frac{h}{b}$, let α =$\;\frac{Z_{se}}{2Z_{0}}$, then it can be simplified as the following formula:

(6) $$\alpha =\frac{h}{2b}\left[\frac{1+\Gamma_{s}e^{-2j\beta l}}{1-\Gamma_{s}e^{-2j\beta l}}\right]$$

The input impedance of CTS radiation stub is $Z_{L}=Z_{se}+Z_{0}$, the following formula can be utilized to calculate the reflection coefficient:

(7) Γ=S11=Zse+Z0−Z0Zse+Z0+Z0=Zse(2Z0)Zse(2Z0)+1=α1+α

The effective coupling coefficient ${\left|K\right|}^{2}$, which represents the energy radiated from the stub, can be expressed as:

(8) $${\left|K\right|}^{2}=1-\frac{1+{\left|\alpha \right|}^{2}}{{\left|1+\alpha \right|}^{2}}$$

The formula shows that the coupling coefficient ${\left|K\right|}^{2}$ of the CTS radiation stub can be adjusted by different values of the width of the radiation stub *h*, and the height of the SIW *b*.

**Figure 5:**
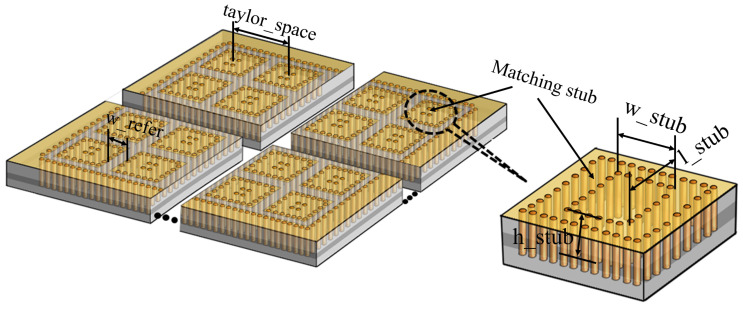
Configuration of the proposed radiation stub.

The CTS radiator using a substrate-integrated waveguide contains three layers (layers 5–7), as shown in Figure 5. It contains 22 × 22 SIW-CTS units and the different thickness of layer 5 (Rogers 4835, $\varepsilon_{r}$ = 3.55, tanδ = 0.0037) and layer 7 (Rogers 4835, $\varepsilon_{r}$ = 3.55, tanδ = 0.0037) is 0.508 mm, and for layer 6 (Rogers 4450F, $\varepsilon_{r}$ = 3.7, tanδ = 0.004) is 0.202 mm.

More specifically, the width of the radiation stub and the distance between the two stubs are w_refer = 0.8 mm, and taylor_space = 6.65 mm, which is designed to meet the Taylor distribution, to obtain low side lobes. Each CTS unit is formed by a metallic layer on the top and 20 via holes arranged in a rectangular shape, as shown in Figure 5. In the center of the unit, another four via holes are made as well. As the plane waves, which are transformed from the spherical waves, propagate along the CTS array, they radiates with tilted beams. Particularly for H-polarization, waves excited from horn antennas 1 & 2 have beam scanning to ±10° along the *x*-axis, while the beams scan to ±10° along the *y*-axis for horn antennas 3 & 4.

**Figure 6:**
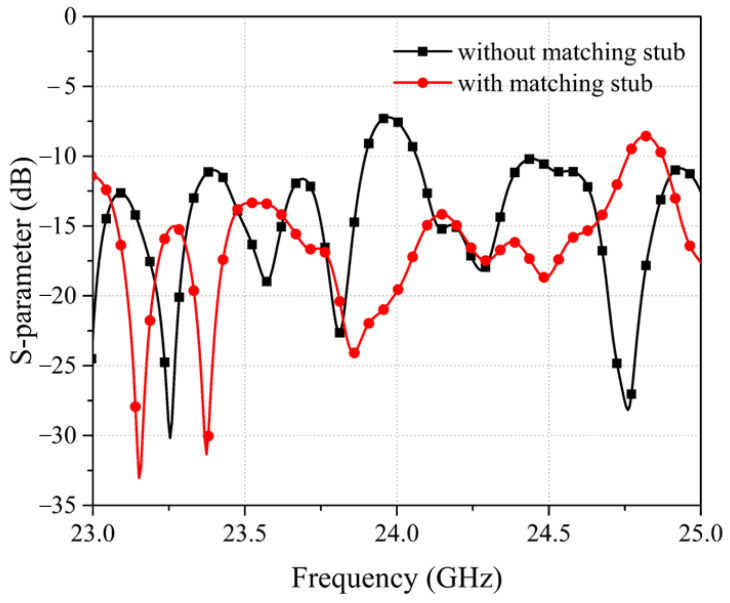
S-parameter of the radiation stub with or without matching stub.

Since it is difficult to make the last radiation stub radiate efficiently due to there being no impedance match stub, the radiation efficiency and gain can be reduced. To overcome the problem, matching stubs have been designed beside each radiation stub. Figure 6 shows the S_11_ simulation results of the radiation stub with or without matching stub. After optimization, the size of the matching stubs is obtained, with w_stub = 0.1 mm, l_stub = 0.1 mm, and h_stub = 1.218 mm, which represent the width, length, and height, respectively.

### 2.4. Simulations

**Figure 7:**
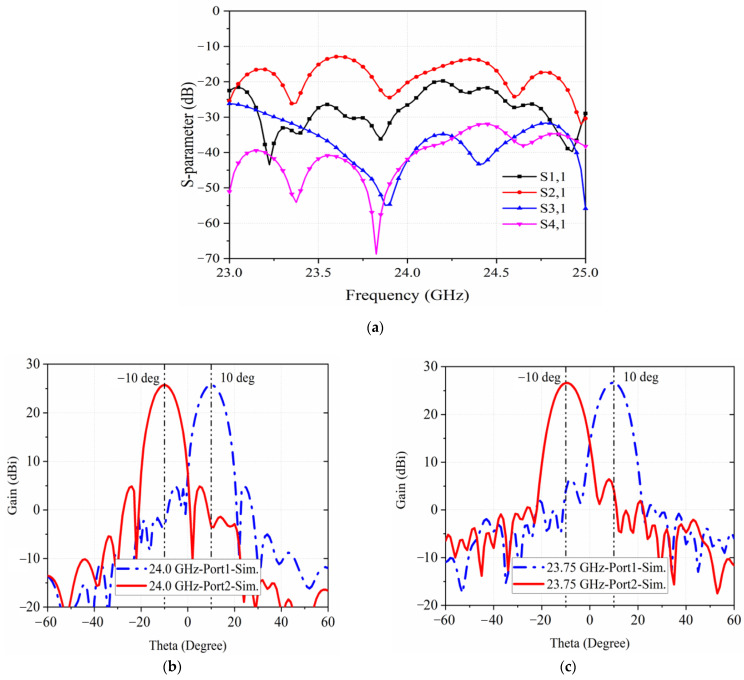
Simulated S11 in (**a**) and radiation patterns in (**b**,**c**) at 24 GHz & at 23.75 GHz.

The proposed antenna is simulated with the commercial software CST. The simulated S_11_ parameter in Figure 7a demonstrates a wide bandwidth from 22–26 GHz. In addition, it has a good isolation between the four ports, because of the symmetry of the antenna. As shown in Figure 7b,c, beams are deflected ±10° by switching between different ports, which can be applied to the wind profile radar. The two radiation beams have the same performance with half-power beam widths of the E-plane and H-plane of the antennas being 8°and 5°. By applying the Taylor distribution to the CTS array, the sidelobe levels of the E-plane and H-plane are −20 and −25 dB, respectively.

## 3. Experimental Implementation and Measurements

**Figure 8:**
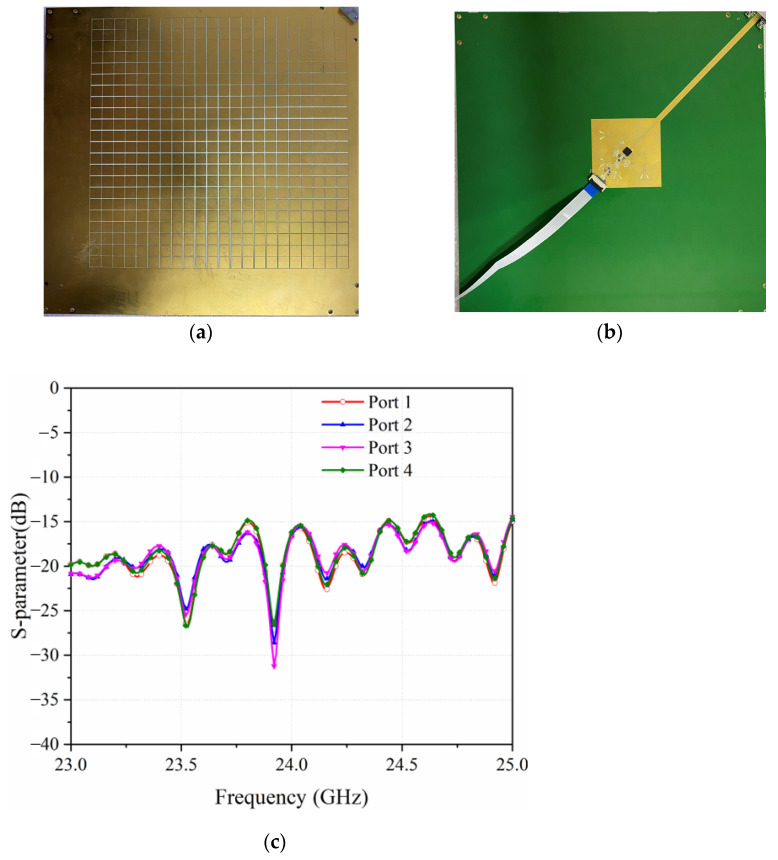
Fabricated samples in (**a**) Top view and (**b**) Bottom view. (**c**) Measured S11 of all the four ports.

**Figure 9:**
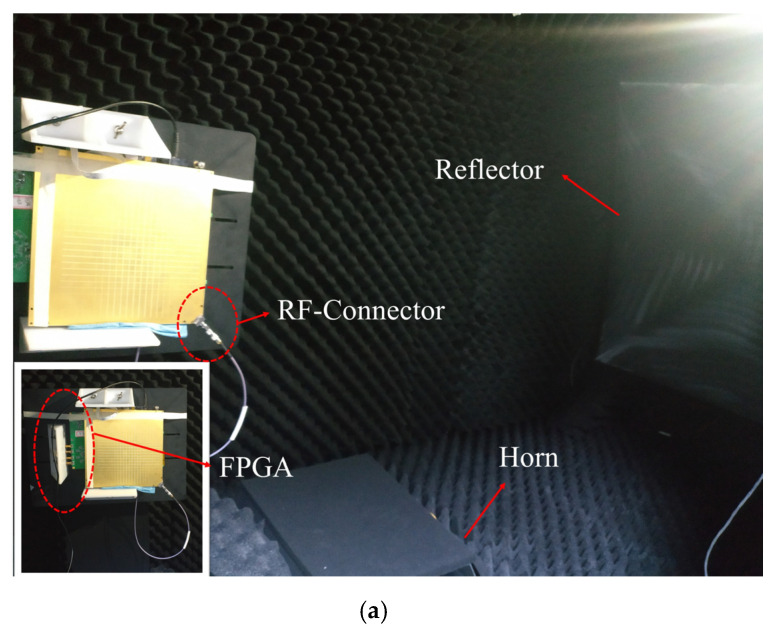

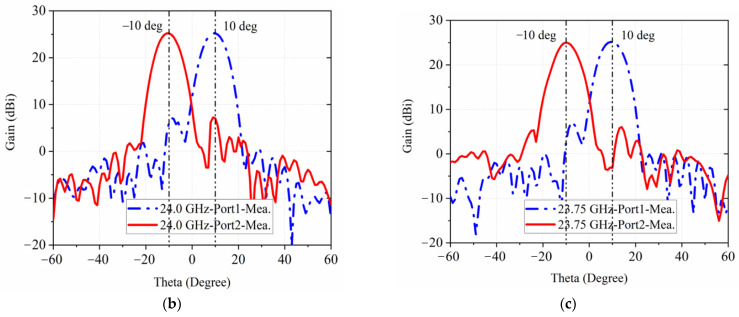
Photograph of the measurement environment in (**a**). Measured radiation patterns in (**b**,**c**) at 24 GHz & at 23.75 GHz.

The sample is fabricated exactly as per the model in Figure 1a, with the PCB technique, as shown in Figure 8a,b. The active antenna utilizes ADRF5045 as the RF switches to switch among the four horn antennas in both ±45° polarizations. As shown in Figure 9a, the proposed antenna is measured in the commercial standard reflector compact range for its high and low sidelobes. For the measurement, the antenna is connected to FPGA board with a cable to control these switches. Meanwhile, use the 2.92 mm end launch RF-connector from SOUTHWEST MICROWAVE to connect the antenna to the microwave anechoic chamber. As shown in Figure 8c, the active antenna operates in the bandwidth from 23–25 GHz with all the four reconfigurable states. As shown in Figure 9b,c, the beams scan to ±10° in H-polarization, working at 24 GHz and 23.75 GHz, respectively. Correspondingly, the relative measured gains are around 25 dBi.

Table 1 compares some key performances of the design in this communication with other related CTS antennas. Compared with the traditional waveguide structures [13,16], the proposed antenna adopts SIW technology and is easy to integrate with the sensor RF front-end. Compared with the previous CTS antenna, the proposed antenna still maintains a lower profile, although it has four beams and has the advantages of dual polarization, large scanning range, high gain, and low side lobes. The proposed reconfigurable four-beam CTS antenna can be well applied to the wind profile radar.

## 4. Conclusions

A high gain, low-profile, multi-beam and dual polarization continuous transverse stub array, operating at 24 GHz, for a radar sensor, is proposed in this communication. The SIW-CTS array is obtained by combining the LSG, coupling layer and radiator together. The LSG contains four H-plane horns and two reflectors, which can generate mutually orthogonal beams with a ±10° deflection. Based on the application scenario of the millimeter wave wind profile, the scanning range is 10–15°, but it is a point-frequency working mode, which is different from frequency-dependent scanning. By applying the Tyler distribution technique to the LSG and radiator, low sidelobes are obtained at the E-plane and the H-plane. The proposed antenna is simulated, fabricated, and measured as well, validating the design procedure. The −10 dB impendence band of the proposed antenna is 22–26 GHz, and the peak gain (24 GHz) is 25.8 dBi with the sidelobe of −20 dB and −25 dB at the E-plane and H-plane, respectively. By controlling the switch chip to excite the four ports, four beams are obtained with a ±10° deflection, which can be utilized for wind profile radars.

## Figures and Tables

**Table 1:** Performance comparison of CTS antenna arrays.

Ref.	Type	Frequency (GHz)	Gain (dBi)	Side Lobe (dB)	Size	Polarization	Scanning Range (°)
[13]	WG	26~40	>26.8	<−12.1	126.5 × 79 × 30 mm^3^ $13.9\;\times \;8.7\;\times \;3.3\;\lambda_{0}{}^{3}$	Single	N\A
[14]	SIW	24.5~29.5	>20.6	<−12.2	40 × 37 × 5.9 mm^3^ $3.6\;\times \;3.3\;\times \;0.5\;\lambda_{0}{}^{3}$	Single	N\A
[15]	SIW	17.2~23.9	<22.6	−15	149.3 × 122 × 4.1 mm^3^ $10\;\times \;8.1\;\times \;0.3\;\lambda_{0}{}^{3}$	Single	N\A
[16]	WG	26~42	>22.9	<−12.6	133 × 93 × 21 mm^3^ $15.1\;\times \;10.5\;\times \;2.4\;\lambda_{0}{}^{3}$	Single	−56.2~−2.2
[17]	SIW	11.8~14.2	<20.6	<−12.2	226 × 103 × 6 mm^3^ $9.8\;\times \;4.5\;\times \;0.3\;\lambda_{0}{}^{3}$	Single	±35
[18]	SIW	36~39	>15.8	<−12	136 × 100 ×N\A mm^3^ $17\;\times \;12.5\;\times \;N\backslash A\;\lambda_{0}{}^{3}$	Single	±30
This work	SIW	22~26	>25	<−20	180 × 180 ×2 5 mm^3^ $14.4\;\times \;14.4\;\times \;2\;\lambda_{0}{}^{3}$	Dual	Reconfigurable four beams in ±10

WG: waveguide; SIW: substrate-integrated-waveguide. $\lambda_{0}$ is the free space wavelength at the center operating frequency.

## Data Availability

Not applicable.
